# US Physicians’ Self-reported Discussions About Tobacco-Free Nicotine Pouches During Clinical Encounters With Patients in 2021

**DOI:** 10.1001/jamanetworkopen.2023.13583

**Published:** 2023-05-16

**Authors:** Mary Hrywna, Michelle T. Bover-Manderski, Olivia A. Wackowski, Michael B. Steinberg, Cristine D. Delnevo

**Affiliations:** 1 Rutgers Center for Tobacco Studies, Rutgers Biomedical and Health Sciences, New Brunswick, New Jersey; 2 Department of Health Behavior, Society and Policy, Rutgers School of Public Health, Piscataway, New Jersey; 3 Department of Biostatistics and Epidemiology, Rutgers School of Public Health, Piscataway, New Jersey; 4 Department of Medicine, Rutgers Robert Wood Johnson Medical School, New Brunswick, New Jersey

## Abstract

This survey study assesses the extent to which physicians discussed tobacco-free nicotine pouches during clinical encounters with patients.

## Introduction

By 2020, major US tobacco manufacturers launched or acquired tobacco-free nicotine pouch brands and sales increased rapidly.^1^ Like smokeless tobacco (SLT), tobacco-free nicotine pouches orally deliver nicotine but, unlike SLT, contain no tobacco leaf. Although these products are not authorized to carry claims of cessation or reduced harm, nicotine pouches contain low levels of toxicants similar to nicotine replacement therapies (NRTs).^2^ Awareness is low, and use of nicotine pouches is modest, but use and interest are higher among those with smoking cessation attempts or plans.^3,4^ Because patients often ask physicians about e-cigarettes for smoking cessation^5^ and nicotine pouches are similar to NRT products, this survey study assessed the extent to which physicians discussed nicotine pouches with patients.

## Methods

We conducted a national cross-sectional survey study from May to October 2021 among a random sample of 500 board-certified physicians from each of 5 specialties in the American Medical Association Physician Masterfile: family medicine, internal medicine, pulmonology, cardiology, and psychiatry. We mailed a study invitation, with instructions for accessing the survey online and a $25 upfront incentive, and up to 3 reminders. The survey assessed physicians’ knowledge, perceptions, and communication about tobacco and nicotine product use and cessation treatment. The study was exempted by the Rutgers Biomedical Health Sciences institutional review board because it involved standard survey procedures and the information obtained was recorded in such a manner that human participants could not be identified, directly or through identifiers linked to participants, with a waiver of written informed consent because risk from participation was minimal and the only record linking participants to their participation in the study would be a signed consent document. This study followed the AAPOR reporting guideline.

The overall response rate was 44.6%. Our outcome of interest was number of physicians ever being asked by patients about tobacco-free nicotine pouches. We also conducted a content analysis of open-ended responses to a question asking physicians to describe their conversations with patients about nicotine pouches. Based on collaboratively identified themes, 3 coders (M.H., M.B.S., and C.D.D.) independently coded all comments, initially achieving 94% agreement; discrepancies were discussed to reach consensus. Data were weighted to adjust for differing probabilities of selection by specialty. Analysis was performed in September 2022 using SAS, version 9.4 (SAS Institute Inc).

## Results

Of 745 participating physicians, 63 (9.7%) reported being asked by patients about tobacco-free nicotine pouches (Table). Discussions were most common for physicians younger than 46 years (17 [15.5%]), those specializing in family medicine (22 [13.2%]), and those using Public Health Service Clinical Practice Guidelines for tobacco treatment (16 [12.7%]).

**Table.** Demographic Characteristics of Physicians and Prevalence of Patient Prompting About Tobacco-Free Nicotine Pouches, 2021^a^

Characteristic	Sample distribution, No.	Prevalence of ever being asked about nicotine pouches by patient, No. (%) [95% CI]^b^
Overall	745	63 (9.7) [7.0-12.5]
Age, mean (95% CI), y	54.8 (53.3-55.7)	NA
Quartile 1 (<46 y)	158	17 (15.5) [7.8-23.1]
Quartile 2 (46-53 y)	149	13 (8.1) [2.7-13.6]
Quartile 3 (54-61 y)	170	10 (7.4) [2.2-12.5]
Quartile 4 (≥62 y)	204	16 (9.3) [4.1-14.5]
Missing	64	NA
Gender		
Male	492	43 (11.2) [7.5-15.0]
Female	211	15 (6.9) [2.7-11.1]
Missing	42	NA
Race and ethnicity		
Non-Hispanic White	459	43 (11.9) [8.0-15.7]
Non-White or Hispanic^c^	241	16 (6.6) [2.6-10.6]
Missing	45	NA
Specialty		
Family medicine	167	22 (13.2) [8.0-18.3]
Internal medicine	142	10 (7.0) [2.8-11.3]
Pulmonology	152	13 (8.6) [4.1-13.0]
Cardiology	145	8 (5.5) [1.8-9.2]
Psychiatry	139	10 (7.2) [2.9-11.5]
Missing	0	NA
Medical school location		
US	544	49 (11.0) [7.6-14.4]
Elsewhere	170	11 (7.0) [1.9-12.0]
Missing	31	NA
PHS clinical practice guidelines		
Unaware	209	18 (11.1) [5.7-16.6]
Aware, not read	318	24 (8.6) [4.6-12.6]
Read, not used	47	3 (6.5) [0.0-16.5]
Used	136	16 (12.7) [5.5-19.9]
Missing	35	NA
Physician’s risk continuum beliefs		
All forms of tobacco equally harmful	408	34 (9.5) [5.8-13.1]
Cigarettes are the most dangerous	330	29 (10.4) [6.1-14.7]
Missing	7	NA

Abbreviations: NA, not applicable; PHS, Public Health Service.

^a^ Question: “These are new tobacco-free oral nicotine products that come in preportioned pouches and are sold under brand names such as Zyn, Velo, On!, and Rogue. Because the nicotine is derived from tobacco, these products are categorized as smokeless tobacco products in the US and are not approved as nicotine replacement therapy or tobacco cessation aids [product images provided]. Have your patients ever asked you about tobacco-free nicotine pouches? (Y/N)”.

^b^ Sample frequencies are unweighted; percentages and 95% CIs are weighted.

^c^ Includes Asian or Pacific Islander, non-Hispanic Black, South Asian, or other.

Fifty respondents described patient discussions about nicotine pouches, which clustered into 3 themes: discouraging use of pouches (20 [40.0%]), learning about nicotine pouches from patients or neutral communication (19 [38.0%]), and communicating to patients that they were open to pouch use for cessation or harm reduction (11 [22.0%]) (Box).

Box. **Major Themes in Conversations With Patients About Tobacco-Free Nicotine Pouches, 2021 (N = 50)^a^**

**Theme: discouraged use (20 [40.0%])**

“Few patients have asked general questions like ‘Would you recommend use of these products?’ Since I know of no data that show they are beneficial I do not recommend them.”
“I advised him to quit. It had nicotine in it and thus I do not recommend it. Patient thought it was safer. I have no data, but I advised him to quit.”
“Tobacco itself is still potentially carcinogenic and so I do not recommend these products nor do I believe them to be safe.”

**Theme: learning about product from patient or neutral (19 [38.0%])**

“They pulled it out of their purse, I googled it.”
“I was not aware that existed until my smokeless tobacco–using patient utilized the product to discontinue use of smokeless tobacco product (chew).”
“Asked as part of history about use.”

**Theme: open to use (11 [22.0%])**

“Recommend to use as a transition to getting off cigarettes.”
“I have at times recommended the switch to smokeless tobacco to my smoking patients with underlying lung disease in an effort to get them to quit smoking.”
“They can help break the physical urge to light and smoke a cigarette, avoiding the ritual and causing less interruption in a person’s daytime activity.”

^a^
Question: “Please describe the conversation(s) you had with your patient(s) about tobacco-free nicotine pouches.”

## Discussion

We found that physicians were asked about tobacco-free nicotine pouches by patients. With market growth, patient prompting about nicotine pouches will likely increase. Previous guidance on e-cigarettes may be helpful to inform physicians’ approach to nicotine pouches—in a discussion of cigarette substitutes, clinicians should urge patients to quit or reduce combustible tobacco use, and while the effects of long-term nicotine pouch use remain unknown, such products are likely less harmful than combustible tobacco.^2,6^ Physicians should ask patients about any tobacco or nicotine use, helping patients differentiate among available products, including tobacco-free nicotine pouches and NRT products approved for cessation.

Study limitations include the potential for reporting bias; prevalence may be underestimated if physicians did not recall or report being asked about nicotine pouches. Although the sample was randomly drawn from a national frame, we did not study all medical specialties. Finally, our sample was small, resulting in wide 95% CIs, and prevalence of nicotine pouch use is still low. However, there were no significant differences in survey response rate by age or gender. Given the continued increase in sales of nicotine pouch products and our observations that patients are asking physicians about them, continued monitoring of physician perceptions and practices around nicotine pouch products is warranted.

## Notes

Supplement.
Data Sharing Statement
