# Supplementary material for: US Physicians’ Self-reported Discussions About Tobacco-Free Nicotine Pouches During Clinical Encounters With Patients in 2021
Source: JAMA Netw Open. 2023 May 16;6(5):e2313583. doi: 10.1001/jamanetworkopen.2023.13583 (PMC10189559; doi:10.1001/jamanetworkopen.2023.13583)
Supplement: Supplement. — Data Sharing Statement [file jamanetwopen-e2313583-s001.pdf]

Data Sharing Statement

Hrywna. US Physicians' Self-reported Discussions About Tobacco-Free Nicotine Pouches During Clinical Encounters With Patients in 2021. *JAMA Netw Open*. Published May 16, 2023. doi:10.1001/jamanetworkopen.2023.13583

Data
Data available: No
